# Human Recombinant Arginase I [HuArgI (Co)-PEG5000]-Induced Arginine Depletion Inhibits Colorectal Cancer Cell Migration and Invasion

**DOI:** 10.3390/ijms20236018

**Published:** 2019-11-29

**Authors:** Houssam Al-Koussa, Maria Al-Haddad, Ralph Abi-Habib, Mirvat El-Sibai

**Affiliations:** Department of Natural Sciences, School of Arts and Sciences, Lebanese American University, 1102-2801 Beirut, Lebanon; houssam.alkoussa@lau.edu (H.A.-K.); maria.alhaddad@lau.edu (M.A.-H.); ralph.abihabib@lau.edu.lb (R.A.-H.)

**Keywords:** colorectal cancer, HuArgI(Co)-PEG5000, arginase, RhoA, migration, invasion, adhesion

## Abstract

Purpose: Colorectal cancer (CRC) is the third most common type of cancer worldwide, and it represents over half of all gastrointestinal cancer deaths. Knowing that cancer cells have a high proliferation rate, they require high amounts of amino acids, including arginine. In addition, several tumor types have been shown to downregulate ASS-1 expression, becoming auxotrophic for arginine. Therefore, Arginine deprivation is one of the promising therapeutic approaches to target cancer cells. This can be achieved through the use of a recombinant human arginase, HuArgI(Co)-PEG5000, an arginine degrading enzyme. Methods: In this present study, the cytotoxic effect of HuArgI(Co)-PEG5000 on CRC cell lines (HT-29, Caco-2, Sw837) is examined though cytotoxicity assays. Wound healing assays, invasion assays, and adhesion assays were also performed to detect the effect on metastasis. Results: Wound healing and invasion assays revealed a decrease in cell migration and invasion after treatment with arginase. Cells that were treated with arginase also showed a decrease in adhesion, which coincided with a decrease in RhoA activation, demonstrated though the use of a FRET biosensor to detect RhoA activation in a single cell assay, and a decrease in MMP-9 expression. Treating cells with both arginase and L-citrulline, which significantly restores intracellular arginine levels, reversed the effect of HuArgI(Co)-PEG5000 on cell viability, migration, and invasion. Conclusion: We can, therefore, conclude that colorectal cancer is partially auxotrophic to arginine and that arginine depletion is a potential selective inhibitory approach for motility and invasion in colon cancer cells.

## 1. Introduction

Colorectal cancer (CRC) is the third most common type of cancer worldwide with approximately 600,000 annual deaths and 1.2 million new cases every year [1]. Colorectal cancer originates from the epithelial lining, most often as a consequence of mutations in the Wnt signaling pathway, in tumor suppressors, in apoptotic genes, and in oncogenes [2,3]. Signs and symptoms of colorectal cancer, as well as its treatment, greatly depend on its location and ability to metastasize [4].

Cell migration is an essential part of cancer invasion and metastasis [5]. For the cell to actively migrate, it follows a well-choreographed series of events [6], which start in response to a chemoattractant. The cell then extends actin-rich protrusions towards the direction of the chemoattractant [7]. The steps that follow for the cell to move forward, include forming adhesion structures to stabilize the protrusion at the leading edge, developing contraction to pull the cell body forward, and finally releasing adhesion structures at the cell rear to retract the cell towards the direction of motility [8]. These processes are regulated by the Rho family of small guanosine triphosphatases (GTPases), which includes key enzymes that play a major role in the reorganization of the actin cytoskeleton [9,10]. Rho GTPases are small monomeric G proteins with a molecular mass of 20 to 40 kDa that belong to the Ras superfamily of proteins [11]. The thee most characterized and studied members of the Rho family are RhoA, Rac1, and Cdc42 [12,13,14]. RhoA has been well characterized and established to regulate the formation of actin–myosin filaments, therefore it regulates the formation of cell adhesion structures needed during the cell motility cycle [12,13,15,16,17].

L-Arginine is a semi-essential amino acid synthesized from L-citrulline via the urea cycle enzymes, argininosuccinate synthetase-1 (ASS-1), and argininosuccinate lyase [18]. The need for arginine depends mainly on the proliferation rate of the cell, hence, cancer cells have a higher requirement for arginine with their survival and proliferation depending on the availability of abundant extracellular arginine reserves [19]. In addition, several tumor types have been shown to lack ASS-1 expression rendering them completely auxotrophic for arginine [20]. Therefore, arginine deprivation, which can be achieved using the human enzyme L-arginase, is a potentially novel, potent, and selective cancer therapeutic approach. Native human L-arginase I (HuArgI), which is conjugated to two Mn^2+^ ion cofactors, has a low saturation constant and a short serum half-life due to the rapid loss of the Mn^2+^ ions in serum [20]. Replacing the Mn^2+^ ions of HuArgI with 2 Co^2+^ ions induces a significant increase in activity and generates an enzyme [HuArgI (Co)], with a significantly higher stability in serum [21]. Addition of polyethylene glycol to [HuArgI (Co)] generates a pegylated human arginase coupled to Co^2+^, [HuArgI (Co)-PEG5000], with an improved catalytic activity, a higher serum stability and a reduced immunogenicity [22,23,24].

We and others have demonstrated the cytotoxicity and selectivity of [HuArgI (Co)-PEG5000]-induced arginine deprivation in a number of tumor types including acute lymphoid leukemia (ALL), hepatocellular carcinoma, glioblastoma multiorme (GBM), and acute myeloid leukemia (AML) [25,26,27,28,29]. The impact of [HuArgI (Co)-PEG5000]-induced arginine deprivation on tumor cell invasion and metastasis has recently been studied in gastric tumors and glioblastoma multiforme (GBM) [30,31]. In this study, we investigate the effect of arginine deprivation on cell migration and invasion in colon cancer and we examine the potential actin regulators involved in this inhibition.

## 2. Results

### 2.1. CRCs Are Sensitive to Arginine Depletion Induced by [HuArgI (Co)-PEG5000]

**Figure 1:**
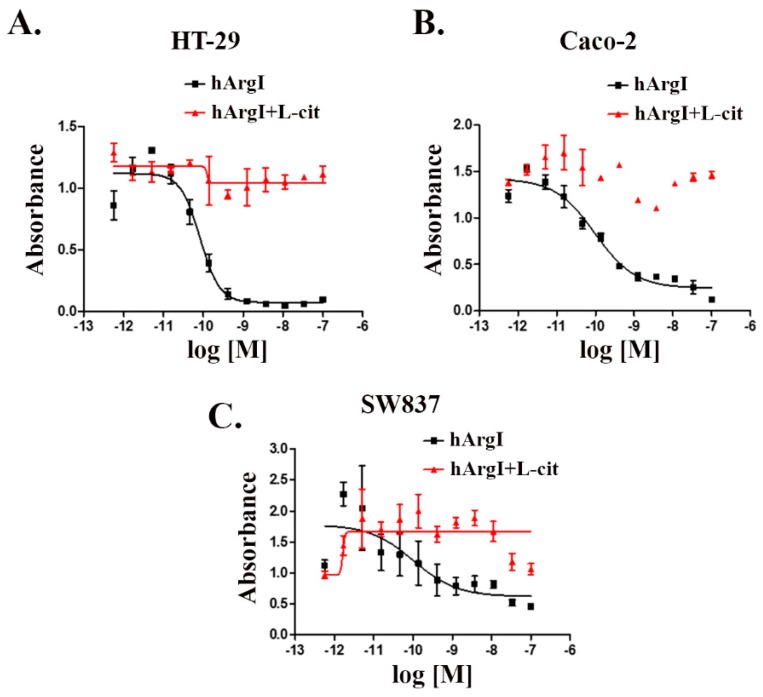
HuArgI(Co)-PEG5000 decreased viability of CRC cells. Non-linear regression curve of the cytotoxicity of the indicated CRC cell lines, HT-29 (**A**)**,** Caco-2 (**B**), and SW837 (**C**) treated with HuArgI(Co)-PEG5000 alone (square) at concentration ranging from 10^−12^ to 10^−7^ M or with L-citrulline (11.4 mM) (triangle) at time 72 h. Data are the mean ± SEM. *p* < 0.05 indicates a statistically significant difference.

We tested the effect of [HuArgI(Co)-PEG5000]-induced arginine deprivation on a panel of CRC cell lines (five cell lines). Three of these cell lines were sensitive to treatment, with IC_50_ values of 83, 97, and 102 pM for HT29, Caco-2, and SW837, respectively at 72 h post treatment (Figure 1). The addition of excess, exogenous L-citrulline, which restores intracellular levels of arginine, led to the rescue of all thee cell lines from arginine deprivation-induced cytotoxicity, demonstrating that they are partially auxotrophic for arginine (Figure 1). This will be used throughout the study as a reversal experiment for the effect of [HuArgI(Co)-PEG5000]-induced arginine deprivation on the behavior of CRC cells in order to establish specificity of the effect observed. In addition to these cell lines, we also examined the effect of [HuArgI(Co)-PEG5000]-induced arginine deprivation on Skco-1 and SW1116 (Supplemental Figure S1). These cells were not considered for the remaining experiments since Skco-1 cells showed moderate sensitivity to arginine depletion and SW1116 cells were not sensitive to arginine depletion, making them less useful in further experiments (Supplemental Figure S1A) (Supplemental Figure S1B).

### 2.2. Arginine Depletion Decreased Colon Cancer Cell Motility

**Figure 2:**
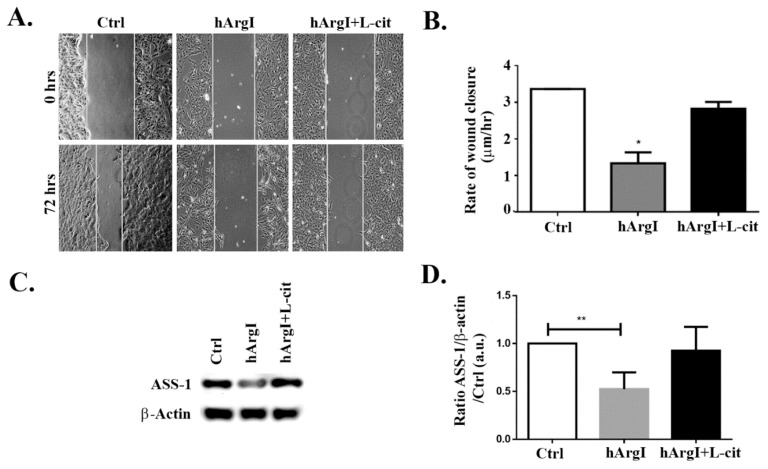
HuArgI(Co)-PEG5000 inhibits motility in CRC cells. Caco-2 cell were treated with HuArgI(Co)-PEG5000 (100 pM) with or without L-citrulline (11.4 mM). Cell monolayers were wounded and images were taken at time 0 h and 72 h. (**A**) Representative wound closure images. The scale bar is 100 μm. (**B**) Frames were quantitated using ImageJ (National Institutes of Health, MA, USA). The width of each wound was measured at 11 different points. The average rate of wound closure was calculated and expressed as µm/h. (**C**) Caco-2 cells were untreated or treated with HuArgI(Co)-PEG5000 with or without L-citrulline for 72 h. Cells were then lysed and blotted for ASS-1 and Actin. (**D**) Quantitation of (**C**) using imageJ software, expressed as the fold change to control. Data are the mean ± SEM. *p* < 0.05 (* *p* < 0.05 and ** *p* < 0.005) indicates a statistically significant difference.

To study the effect of [HuArgI(Co)-PEG5000]-induced arginine deprivation on the migration of CRC cells, wound healing assays were performed. HT-29, Caco-2, and Sw837 cell lines were allowed to grow in low serum medium in monolayers, left untreated or treated with HuArgI(Co)-PEG500 with or without L-citrulline for 72 h. The results showed that [HuArgI(Co)-PEG5000]-induced arginine deprivation led to a significant decrease in the rate of wound closure when compared to that of untreated cells, in Caco-2 cells (*p* = 0.0257) (Figure 2A,B). The addition of excess L-citrulline completely reversed the effect of HuArgI(Co)-PEG5000] on the rate of wound healing, generating a rate largely similar to that of untreated cells (Figure 2A,B). This indicates that [HuArgI(Co)-PEG5000]-induced arginine deprivation leads to a significant decrease in CRC cell motility. We also tested the effect of HuArgI(Co)-PEG5000 treatment on HT-29 and SW837 cells (Supplemental Figure S2). While both cell lines showed a decrease in wound closure in response to arginase treatment, the inhibitory effect on SW837 motility was less pronounced (Supplemental Figure S2C,D) and that on HT-29 motility was not reversed with L-citrulline addition (Supplemental Figure S2A,B). For these reasons, we continued the study with Caco-2 cells, which showed sensitivity to arginine depletion with regards to their cytotoxicity and migration and which were also responsive to reversal after L-citrulline treatment (partial auxotrophy) making them a very useful model for this study. When Western blots were performed in these cells, they showed a high basal expression of ASS-1 (Figure 2C,D), which is further proof for partial auxotrophic. Interestingly as well, the cells showed a decrease in ASS expression in the [HuArgI(Co)-PEG5000]-treated cells. This was reversed with L-citrulline addition.

### 2.3. Arginine Depletion Decreased Colon Cancer Cell Adhesion to Collagen

**Figure 3:**
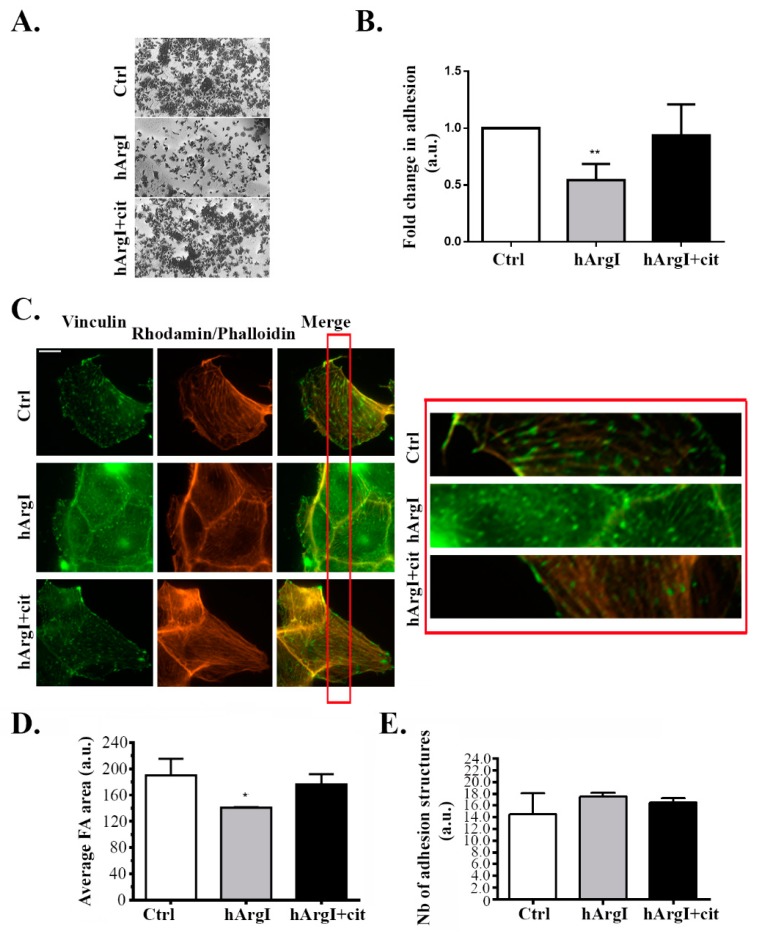
HuArgI(Co)-PEG5000 decreased cell adhesion in Caco-2 cells. (**A**) Representative micrographs of Caco-2 cells plated at the different indicated conditions, fixed and stained with crystal violet as described in the methods. Scale is 100 μm. (**B**) Quantitation of (**A**) expressed as fold difference to control. Absorption of the solubilized Crystal violet was measured at 550 nm using an ELISA plate reader. (**C**) Representative micrographs of Caco-2 cells untreated or treated with HuArgI (Co)-PEG5000 (100 pM) with or without L-citrulline (11.4 mM) and stained with anti-vinculin. Cells were imaged using a 60× objective. Scale is 10 μm. The red inset indicates a region in the cell magnified 10X in the right panel to better show the FAs. (**D**) Quantitation of areas (**E**) or numbers of focal adhesions, Data are the mean ± SEM. *p* < 0.05 (* *p* < 0.05 and ** *p* < 0.001) indicates a statistically significant difference.

To further study the effect of the drug on the cell migration cycle, cell adhesion was examined by performing a cell adhesion assay. Caco-2 cells were plated on collagen left untreated or treated with HuArgI(Co)-PEG5000 with or without L-citrulline. [HuArgI(Co)-PEG5000]-induced arginine deprivation caused a significant decrease in total cell adhesion of approximately 50% compared to controls (*p* = 0.0053). Addition of excess L-citrulline reversed the effect of arginine deprivation, restoring cell adhesion to levels similar to those of control cells (Figure 3A,B). Caco-2 cells were then immunostained for vinculin, a component of adhesion structures and focal adhesions were quantitated with a specific macro according to the manufacturer’s instructions using ImageJ [32,33]. Treatment of Caco-2 cells with HuArgI(Co)-PEG5000 with or without L-citrulline had no significant effect on the number of adhesion structures but appeared to lead to a decrease in the area of these structures, which might be consistent with the loss in adhesion capacity of the cells (loss of maturity of focal adhesions) and might be an indication of a decrease in the activity of RhoA (which regulates the maturation of focal adhesions) [15,17] (Figure 3C–E).

### 2.4. Arginine Depletion Decreased RhoA Activation in Colon Cancer Cells

**Figure 4:**
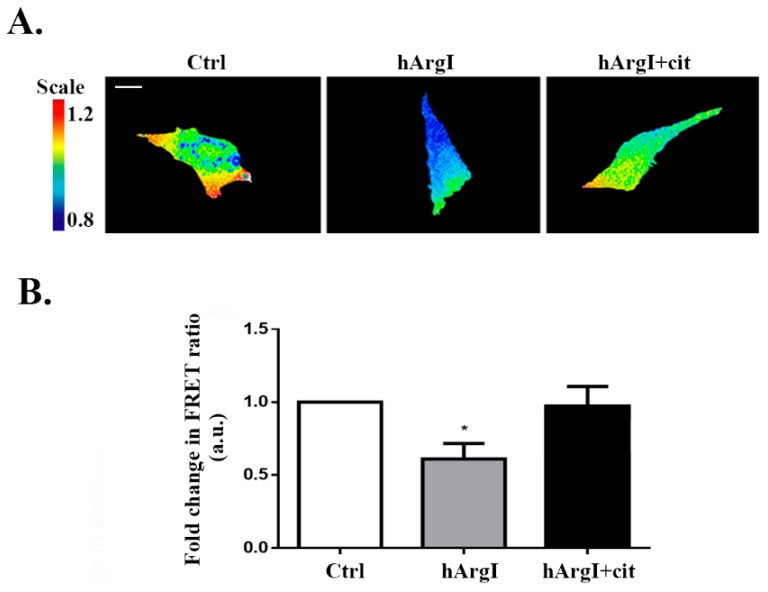
HuArgI(Co)-PEG5000 decreased RhoA activation in CRC cells. (**A**) Representative micrographs of Caco-2 cells transfected with the RhoA FRET biosensors, cells were then untreated or treated with HuArgI(Co)-PEG5000 alone (100 pM) or with without L-citrulline (11.4 mM). The ratiometric images were obtained by normalizing the raw FRET image to the CFP image as described before [13]. Scale is 10 μm. (**B**) Quantitation of (**A**) expressed as fold difference to control. Data are the mean ± SEM. * is *p* < 0.05 indicating a statistically significant difference.

Since RhoA is known to regulate cell adhesion, we then investigated whether the decrease in cell adhesion driven by [HuArgI(Co)-PEG5000]-induced arginine deprivation is mediated though an effect on RhoA activation. The activation levels of RhoA were studied using a previously described unimolecular effector-based RhoA FRET biosensor [13,34]. Caco-2 cells were transfected with the RhoA biosensor and left untreated or treated with HuArgI(Co)-PEG5000 with or without L-citrulline for 72 h. Cells were then imaged for their RhoA activation and the FRET signal was normalized to the total expression of the biosensor. [HuArgI(Co)-PEG5000]-induced arginine deprivation resulted in a significant decrease in RhoA activation levels (approximately 60%), compared to control cells (*p* = 0.0347). Addition of excess L-citrulline reversed the effects of arginine deprivation, restoring RhoA activation almost to control levels (Figure 4A,B).

### 2.5. Arginine Depletion Decreased Colon Cancer Cell Invasion

**Figure 5:**
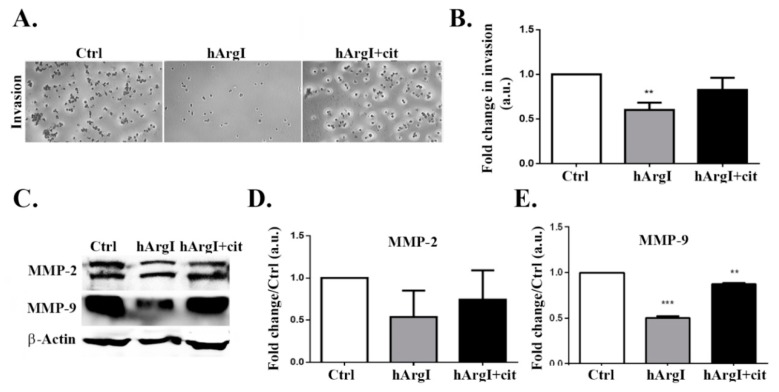
HuArgI(Co)-PEG5000 decreased invasion and MMP expression in CRC cells. (**A**) Representative micrographs of Caco-2 untreated or treated with HuArgI(Co)-PEG5000 (100 pM) without or with L-citrulline (11.4 mM) and subjected to a 72 h cell invasion assay. Scale is 100 μm. (**B**) After cells invaded, they were stained, stain was then extracted and absorbance measured at 560 nm. Data are fold change compared to control ± SEM. (**C**/**D**) Caco-2 cells were untreated or treated with HuArgI(Co)-PEG5000 with or without L-citrulline for 72 h. Cells were then lysed and blotted for MMP-2 (**C**) and MMP-9 (**D**). (**E**/**F**) Quantitation of (**C**) and (**D**) using the imageJ software expressed as fold change from control. Data are the mean ± SEM. *p* < 0.05 (** *p* < 0.001 and *** *p* < 0.0001) indicates a statistically significant difference.

After establishing the effect in 2D cell migration, we were interested in determining its role in 3D cell invasion. The impact of [HuArgI(Co)-PEG5000]-induced arginine deprivation on cell invasion was studied using an in-vitro Matrigel invasion assay with FBS as chemo-attractant. The trans-well chambers were filled with serum free media used as a negative control. Cellular invasion decreased by approximately 40% in cells treated with HuArgI(Co)-PEG5000 compared to control cells (*p* = 0.0011). This was reversed by the addition of excess L-citrulline, which restored cellular invasion to levels comparable to those of control cells (Figure 5A,B).

### 2.6. HuArgI(Co)-PEG5000 Downregulates MMP Expression in the CRC Cell Line

The decrease in cellular invasion was further examined by determining the effect of arginine deprivation on the expression levels of MMP-2 and MMP-9. Caco-2 cells treated with HuArgI(Co)-PEG5000 were plated with or without L-citrulline for 72 h, followed by western immunoblotting. [HuArgI(Co)-PEG5000]-induced arginine deprivation led to a significant decrease only in MMP-9 expression levels (*p* = 0.0008), compared to control cells. The decrease in MMP-9 levels induced by arginine deprivation was reversed by the addition of L-citrulline, which restored MMP-9 back at control levels. Expression levels of MMP-2 were not affected by arginine deprivation and remained similar to control levels (Figure 5C,D).

## 3. Discussion

Arginine deprivation has been used as a cytotoxic approach for the selective targeting of a large number of tumors that are auxotrophic for arginine. However, the impact of arginine deprivation on the motility, invasion and migration of these tumors has not been thoroughly investigated yet. In this study, we investigate the impact of [HuArgI(Co)-PEG5000]-induced arginine deprivation on cell motility, invasion, and migration of colon carcinoma cells. Arginine deprivation using HuArgI(Co)-PEG5000 had a strong cytotoxic effect on CRC cell lines indicating that colon cancer cells are auxotrophic for arginine hence unable to survive in the absence of extracellular sources of arginine. However, we and others have shown the presence of a mixture of completely auxotrophic and partially auxotrophic cells in different tumor types [27,28,35,36,37]. Both types are equally sensitive to arginine deprivation using HuArgI(Co)-PEG5000 with the difference being that completely auxotrophic cells do not express the urea cycle enzyme argininosuccinate synthetase (ASS-1) and are, therefore, completely unable to synthesize arginine while partially auxotrophic cells express ASS-1 and are capable of arginine synthesis but at levels well below their metabolic needs dictated by fast replication rates [18,38,39]. Since they express ASS-1, partially auxotrophic cells are rescued from the effects of arginine deprivation by the addition of excess L-citrulline, which increases the rate of endogenous arginine production to levels compatible with the replicative needs of these cells [40]. In this study, Addition of L-citrulline led to the reversal of arginine deprivation-induced cytotoxicity, indicating that the CRC cell lines used are partially auxotrophic for arginine. The auxotrophy status of these cells provides us with a powerful tool to demonstrate that any effect seen is in fact induced by arginine deprivation since it should be reversed by the addition of L-citrulline.

In this study, we investigated the effect of HuArgI(Co)-PEG5000 treatment on 2D cell motility as well as 3D invasion. Treatment with HuArgI(Co)-PEG5000 led to a decrease in cell migration as evidenced by a wound healing assay. This was consistent with a decrease in cell adhesion observed in response to treatment which would render the cells, while still capable of protruding, unable to fix the newly formed protrusions on the extracellular matrix in order to move forward [15,16]. Indeed, immunostaining analysis of focal adhesion (FA) structures revealed that, while the total number of focal adhesions remains unchanged following treatment, their size appears to be smaller, suggesting that the drug hindered the maturation of FAs, thus impairing cellular adhesion. Point contacts need to mature into stress-relaying focal adhesions at the leading edge of cells in order to enable productive cell motility [13,15,16,41]. This decrease in cell adhesion in response to treatment would account for the decrease in cell motility.

After investigating the role of HuArgI(Co)-PEG5000 on the 2D motility of Caco-2 cells, we had to be determine its effect on 3D invasion. Results of a matrigel based invasion assay indicated that cell invasion decreased significantly upon treatment with HuArgI(Co)-PEG5000. Taking into consideration the fact that matrix metalloproteases (MMPs) degrade the extracellular matrix in order for a cell to invade [42], we performed Western blots in order to examine the expression levels of both MMP-2 and MMP-9. Results showed that the expression of MMP-9 was reduced when cells were treated with HuArgI(Co)-PEG5000, while expression of MMP-2 was not affected, hence indicating that the decrease in the expression of MMP-9 seems to contribute to the observed decrease in cell invasion following treatment.

As it was previously established in the literature, RhoA orchestrates significant mechanisms of cell motility including the retraction of the tail [43,44,45] and the maturation of FAs in order for the cell to adhere [13,15,17,41,46]. This led us to suspect that the effect of treatment with HuArgI(Co)-PEG5000 might be mediated though RhoA. FRET-based biosensor analysis of RhoA activation in these cells showed a substantial decrease in RhoA activation in response to treatment. Hence, by hindering RhoA activation levels, the drug might be affecting all the aforementioned processes. Interestingly, when cells were treated with HuArgI(Co)-PEG5000, rhodamin–phalloidin staining revealed an almost complete lack of stress fiber formation, consistent with a decrease in RhoA activation (Figure 3C). This is consistent with previous reports from our laboratory showing that the knock down of RhoA downregulated cellular adhesion and migration in colon cancer cells [5]. The observed effects of HuArgI(Co)-PEG5000 treatment on cell invasion could be though the decrease in the expression of MMPs or could be a direct effect of the loss of FAs which contribute to matrix degradation [47] or both.

We also observed a decrease in the expression of ASS-1 in response to [HuArgI(Co)-PEG5000]-induced arginine deprivation, suggestive of a potential positive feedback loop between arginine presence and the expression of ASS-1 (Figure 2C,D).

## 4. Materials and Methods

### 4.1. Cell Lines

Human colon carcinoma cell line HT-29 and human colorectal adenocarcinoma cell lines Caco-2, Sw837, Sw1116, and Skco-1 were obtained from the American Type Culture Collection (ATCC) and cultured in DMEM medium supplemented with 10% FBS and 100 U penicillin/streptomycin at 37 °C and 5% CO_2_ in a humidified chamber.

### 4.2. Treatment with Drugs

In all the experiments in this study, L-citrulline was added to cells at a concentration of 11.4 mM. The concentrations of HuArgI(Co)-PEG5000 used for the cytotoxicity assay are indicated below. For all the remaining assays, cells were treated with a concentration of HuArgI(Co)-PEG5000 of 100 pM, which approximately corresponds to its IC_50_ for all the cell lines used, at 72 h post-treatment, according to the values established in the cytotoxicity assay.

### 4.3. Cytotoxicity Assay

Cytotoxicity of HuArgI(Co)-PEG5000 was determined using a proliferation inhibition assay, as described previously [27,28]. Briefly, aliquots of 10^4^ cells/well in 100µL cell culture medium were plated in flat-bottom 96-well plates (Corning Inc. Corning, NY, USA). When used, L-citrulline was added to the cells at a concentration of 11.4 mM. This was followed by the addition of 50 µL HuArgI(Co)-PEG5000 in media to each well (Corning Inc. Corning, NY, USA) to yield concentrations ranging from 10^−7^ to 10^−13^ M. Following a 72-h incubation at 37 °C/5% CO_2_, 50 µL of XTT cell proliferation reagent (Roche, Basel, Switzerland) were added to each well and the plates incubated for 4 h. Absorbance was then read at 450 nm using a Varioskan Flash plate reader (Thermo Fisher Scientific, Waltham, MA, USA). Nominal absorbance and percent maximal absorbance were plotted against the log of concentration and a non-linear regression with a variable slope sigmoidal dose response curve was generated along with IC_50_ using GraphPad Prism 5 software (GraphPad Software, San Diego, CA, USA) (as previously described [29,48]).

### 4.4. Antibodies and Reagents

Pegylated human recombinant Arginase I cobalt [HuArgI (Co)-PEG5000] (Pegzilarginase) was a gift from Aeaglea BioTherapeutics (Aeaglea BioTherapeutics, Austin, TX, USA). The following primary antibodies were used in this study: Rabbit polyclonal anti-beta Actin, mouse monoclonal anti-MMP2[6E3F8], rabbit polyclonal anti-MMP9, rabbit polyclonal anti-ASS-1, and mouse monoclonal anti-vinculin [VIN-54] (Abcam Inc., Cambridge, UK). The following secondary antibodies were used in this study: Alexa Fluor 488 goat anti-mouse IgG (H + L) was obtained from Invitrogen). Anti-rabbit and anti-mouse HRP-conjugated secondary antibodies were obtained from Promega (Promega Co., Madison, WI, USA).

### 4.5. Western Blotting

Whole cell lysates were prepared by scraping the cells with Laemmli sample buffer that contains 4% SDS, 20% glycerol, 10% β mecraptoethanol, 0.004% bromophenol blue, and 0.125 M Tris HCl (pH 6.8). SDS-PAGE was carried out under standard conditions and proteins were blotted onto a PVDF membrane. The membranes were then blocked with 5% bovine serum albumin for 1 h and then incubated overnight at 4 °C with either primary antibody against MMP2 (abcam,1:1000 dilution), MMP9 (abcam, 1:1000), or β-actin (abcam, 1:2500). After the incubation with the primary antibody, the membranes were washed and incubated with secondary antibody at a concentration of 1:1000 for 1 h at room temperature. The membranes were then washed, and the bands visualized by treating the membranes with ECL (GE Healthcare). The levels of protein expression were compared by densitometry using ImageJ software.

### 4.6. Wound Healing Assay

Cells were grown to confluence on culture plates while incubated with [HuArgI (Co)-PEG5000] in the presence and absence of excess L-citrulline. After 24 h, a wound was made in the monolayer with a sterile pipette tip. Cells were then washed twice with PBS to remove debris and new medium was added. Phase-contrast images of the wounded area were captured at 0 and 72 h after wounding. Wound widths were measured at 11 different points for each wound, and the average rate of wound closure was calculated (in μm/h) (described in [49,50]). The assay was done using infinity-corrected optics on a Zeiss Observer Z1 microscope supplemented with a computer-driven Roper cooled CCD camera and operated by Zen software (Zeiss).

### 4.7. Immunostaining

Cells were plated on glass coverslips and incubated with the drug for 72 h. Cells were then fixed with 4% paraformaldehyde for 10 min at 37 °C and permeabilized with 0.5% Triton-X 100 for 15 min on ice. For blocking, cells were incubated with 1% filtered BSA in PBS for 1 h. Samples were then stained with primary antibodies overnight at 4 °C and with fluorophore-conjugated secondary antibodies for 1 h. Fluorescent images were taken using a 63× objective lens on a Zeiss Observer Z1 microscope supplemented with a computer-driven Roper cooled CCD camera and operated by Zen software (Zeiss).

### 4.8. Quantitation of Focal Adhesions and Invadopodia

ImageJ (National Institutes of Health, MA, USA) was used to quantitate focal adhesions or invadopodia, where appropriate. Briefly, two main plugins, CLAHE and Log3D were used to quantitate these structures and the threshold was determined depending on their size. CLAHE enhances the local contrast of the image and Log3D filters the image based on user-predefined parameters that will allow us to detect and analyze focal adhesions Areas of focal adhesions (seen by vinculin staining) in different samples were presented as fold difference to control due to variability among experiments. The number of invadopodia (seen by TKS4 staining) was expressed as absolute values of the means in every sample (from three experiments).

### 4.9. Cell Adhesion Assay

Ninety-six well plates were coated with collagen using Collagen Solution, Type I from rat tail (Sigma-Aldrish, St. Louis, MO, USA) overnight at 37 °C then washed with washing buffer (0.1% BSA in DMEM). The plates were then blocked with 0.5% BSA in DMEM at 37 °C in a CO_2_ incubator for 1 h. This was followed by washing the plates and chilling them on ice. Meanwhile, the cells were trypsinized and counted to 4 × 10^5^ cell/mL. Fifty microliters (50 μL) of cells were added in each well and incubated at 37 °C in a CO_2_ incubator for 30 min. The plates were then shaken and washed three times. Afterwards, cells were fixed with 4% paraformaldehyde at room temperature for 10 min, washed, and stained with crystal violet (5 mg/mL in 2% ethanol) for 10 min. Following the staining with crystal violet, the plates were washed extensively with water and left to dry completely. Crystal violet was solubilized by incubating the cells with 2% SDS for 30 min. The absorption of the plates was read at 550 nm, using a Thermo scientific Varioskan Flash Multimode reader (Thermo Fisher Scientific, Waltham, MA, USA).

### 4.10. Cell Invasion Assay

Cells were grown to confluence on culture plates. After 72 h treatment with the drug, invasion assay was performed using the Matrigel-based invasion assay (Millipore) according to manufacturer′s instructions. Cells were harvested, centrifuged, and then resuspended in quenching medium (without serum). Cells were then brought to a concentration of 1 × 10^6^ cells/mL. In the meantime, inserts were pre-warmed with 300 μL of serum free medium for 30 min at room temperature (Corning, Corning, NY, USA). After rehydration, 250 μL of media was removed from inserts and 250 μL of cell suspension was added. Inserts were then placed in a 24-well plate, and 500 μL of complete media (with 10% serum) was added to the lower wells. Plates were incubated for 24 h at 37 °C in a CO_2_ incubator. Following 48 h of incubation, inserts were stained for 20 min at room temperature with 400 μL of cell stain provided with the kit. Stain was then extracted with extraction buffer (also provided with the kit). One-hundred microliters (100 µL) of extracted stain was then transferred to a 96-well plate suitable for colorimetric measurement using a plate reader. Optical density was then measured at 560 μm.

### 4.11. Fluorescence Resonance Energy Transfer

Caco-2 cells were transfected with 1 μg of the RhoA fluorescence resonance energy transfer (FRET)-based biosensor plasmid [34], which shows an increase in FRET ratio with GTP loading of RhoA, using using Lipfectamine LTX with Plus reagent (Invitrogen, Carlsbad, CA, USA) as described by the manufacturer. The biosensor consists of (from the *n*-terminus) the Rho binding domain (RBD) of the effector Rhotekin, a cyan fluorescent protein (CFP), a protease resistant 17-mer unstructured linker, a yellow florescent protein (YFP) domain, and a full-length RhoA [34]. Activation of RhoA can be determined as a ratio of the FRET image over the CFP image [13]. FRET image sequences were obtained with an automated using a 63× objective lens on a Zeiss Observer Z1 microscope supplemented with a computer-driven Roper cooled CCD camera and operated by Zen software (Zeiss). A CFP/YFP FRET filter cube was used: YFP was imaged with exciter S500/20 and emitter S535/30 (YFP/acceptor image), CFP was imaged with exciter S430/25 and emitter S470/30 (CFP/donor image) or S535/30 (FRET image). Images were background corrected and the YFP images were thesholded to generate a binary mask with values of 1 within the cell and 0 for the background. This was used to remove the background from ratio calculations, by multiplying CFP and FRET images by the mask. The increase in FRET signal due to activation of RhoA was detected by dividing the FRET image (CFP excitation- YFP emission) by the donor image (CFP excitation- CFP emission). FRET signals were quantified by averaging the mean FRET ratio in all the cell area and values were then normalized to control cells (untreated) and expressed as fold change.

### 4.12. Statistical Analysis

The results reported represent the average values of thee independent experiments. All error estimates are given as ± standard error of the mean (SEM). The *p*-values were calculated by *t*-test or one-way analysis of variance (ANOVA) using GraphPad Prism. Results showed statistical significance with *p*-value ≤ 0.05.

## 5. Conclusions

In this study, we have shown that CRC cell lines are partially auxotrophic for arginine and can be selectively targeted using HuArgI (Co)-PEG5000-induced arginine deprivation. In addition, we have demonstrated that arginine depletion also affects cell adhesion, migration and invasion and that this effect is, at least in part, though an inhibition of RhoA activation and a decrease in metalloprotease expression.

## Supplementary Materials

Supplementary materials can be found at https://www.mdpi.com/1422-0067/20/23/6018/s1.
