# Supplementary material for: Human Recombinant Arginase I [HuArgI (Co)-PEG5000]-Induced Arginine Depletion Inhibits Colorectal Cancer Cell Migration and Invasion
Source: Int J Mol Sci. 2019 Nov 29;20(23):6018. doi: 10.3390/ijms20236018 (PMC6929075; doi:10.3390/ijms20236018)
Supplement: Supplementary file 1 [file ijms-20-06018-s001.pdf]

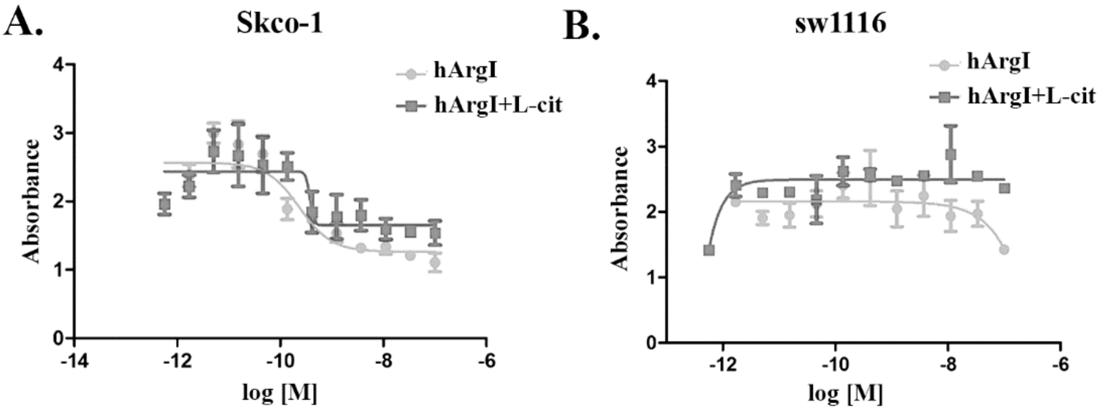

Supplemental Figure S1. HuArgI(Co)-PEG5000 effect on (**A**) Skco-1 and (**B**) sw1116 cells**.** Non-linear regression curve of the cytotoxicity of Skco-1 and sw1116 cells treated with HuArgI(Co)-PEG5000 alone (square) at concentration ranging from 10^{-12} to 10^{-7} M or with L-citrulline (11.4 mM) (triangle) at time 72 h.

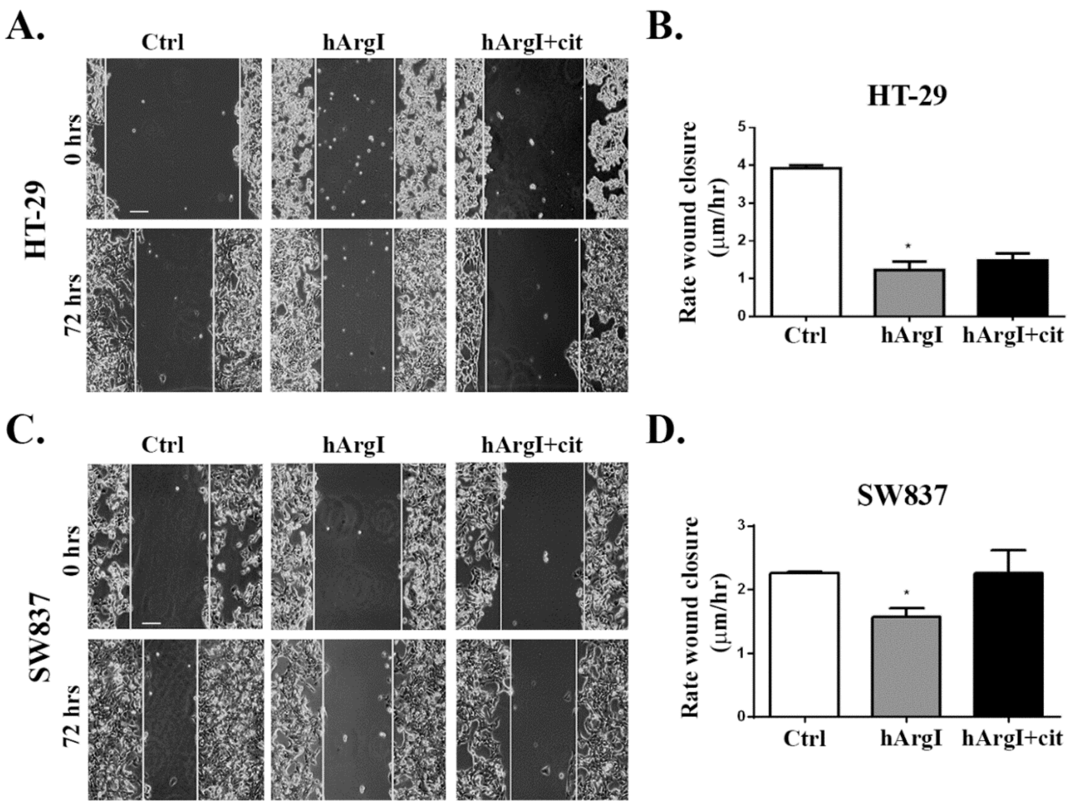

Supplemental Figure S2. HuArgI(Co)-PEG5000 inhibits motility in HT-29 and SW837 cells. HT-29 and SW837 cell lines were treated with HuArgI(Co)-PEG5000 (100 pM) with or without L-citrulline (11.4 mM). Cell monolayers were wounded and images were taken at time 0 hr and 72 h. (**A** and **C**) Representive wound closure of HT-29 (**A**) and Sw837 cells (**C**)**,** respectively. (**B** and **D**) Frames in (**A**) and (**C**) were quantitated using ImageJ. The width of each wound was measured at 11 different points. The average rate of wound closure was calculated and expressed as μm/h. data are the mean ± SEM. $p < 0.05$ indicates a statistically significant difference. * $p < 0.05$. Scale bar is 100 μm.
